# Predictors and outcomes of angioplasty and stenting in acute intracranial atherosclerosis-related vertebrobasilar artery occlusion

**DOI:** 10.3389/fneur.2025.1429931

**Published:** 2025-02-05

**Authors:** Seol Bin Park, Byung Hyun Baek, Yun Young Lee, Seul Kee Kim, Chan Park, Byung Chan Lee, Hyoung Ook Kim, Woong Yoon

**Affiliations:** Department of Radiology, Chonnam National University Medical School, Chonnam National University Hospital, Gwangju, Republic of Korea

**Keywords:** vertebrobasilar artery occlusion, thrombectomy, atherosclerosis, angioplasty, stenting

## Abstract

**Objective:**

This study aimed to investigate clinical outcomes and predictors of favorable functional outcomes after endovascular treatment, including emergent angioplasty and stenting, in patients with intracranial atherosclerotic stenosis (ICAS)-related occlusions in vertebrobasilar arteries.

**Materials and methods:**

This retrospective case series included 46 patients with acute occlusions of vertebrobasilar arteries, due to underlying ICAS. All patients underwent a thrombectomy, followed by angioplasty and/or stenting. We performed logistic regression analyses to identify independent predictors of favorable outcomes. A favorable outcome was defined as a score of 0–3 on the 90-day modified Rankin Scale.

**Results:**

Overall, successful reperfusion was achieved in 44/46 (95.7%) patients and 90-day favorable outcomes were achieved in 21/46 patients (45.7%). The 90-day mortality rate was 23.9% (11/46). In a multivariate binary logistic analysis, independent predictors of favorable outcome were hyperlipidemia (OR = 7.866, 95% CI: 1.093–56.590, *p* = 0.040), admission hyperglycemia (OR = 0.144, 95% CI: 0.023–0.914, *p* = 0.040), basilar artery occlusion (OR = 0.086, 95% CI: 0.008–0.907, *p* = 0.041), and treatment with angioplasty alone (OR = 9.779, 95% CI: 1.022–93.525, *p* = 0.048).

**Conclusion:**

Our findings suggested that emergent angioplasty and/or stenting could yield high rates of successful reperfusion and favorable outcomes in patients with ICAS-related occlusions in vertebrobasilar arteries. Our study also demonstrated that hyperlipidemia and treatment with angioplasty alone were associated with favorable outcomes, in contrast, admission hyperglycemia and basilar artery occlusion were associated with unfavorable outcomes in these patients.

## Introduction

Intracranial atherosclerotic stenosis (ICAS) is an important cause of strokes due to acute large vessel occlusion (LVO) in Asian, African Americans, and Hispanic populations (1). Current endovascular treatment (EVT) with thrombectomy devices, such as a stent retriever and an aspiration catheter, are not effective in achieving recanalization, in most cases of ICAS-related acute LVO (2, 3). The EVT strategy for ICAS-related LVO should be different from that for embolism-related LVO, because the pathophysiologic mechanisms are dissimilar between these two situations. The basic pathophysiologic mechanism of an acute ICAS-related LVO is an in-situ thrombosis, due to rupture of an unstable atherosclerotic plaque. Thus, after a first-line thrombectomy, the ICAS-related LVO requires additional rescue therapy for treating the culprit ICAS lesion. This procedure is essential in achieving recanalization and preventing reocclusion (2, 3). Treatment outcomes, such as recanalization success and procedural complications, are greatly affected by rescue therapies, including angioplasty, stenting, and the administration of antiplatelet drugs in patients with acute ICAS-related LVO.

ICAS-related LVO is more common in the posterior circulation than in the anterior circulation. In Asian countries, among patients with acute basilar artery occlusion, 25–58% had ICAS as the underlying etiology (4, 5). In contrast, 15–20% of acute anterior circulation LVO has been attributed to ICAS in Asian countries (6, 7). Moreover, it has been suggested that an LVO in the posterior circulation that was attributed to ICAS led to worse outcomes than a cardioembolic LVO (8, 9). Despite its clinical significance, few studies have focused on EVT outcomes in patients with acute ICAS-related LVO in the posterior circulation and outcomes varied widely according to treatment methods including rescue therapies (8–13). In a MR CLEAN (Multicenter Randomized Controlled Trial of Endovascular treatment for Acute Ischemic Stroke in the Netherlands) registry data, Pirson et al. found that patients with ICAS had a lower than 33% chance of a favorable outcome (mRS 0–3 at 90 days) compared to those with cardioembolism (8). On the other hand, Sun et al. reported that EVT could achieve relatively high rates of successful recanalization (86%) and favorable outcome (53%) in 116 Chinese patients with acute ICAS-related basilar artery occlusion (11). Otherwise, Lee et al. and Nguyen et al. reported that there was no difference in functional outcomes after EVT between basilar artery occlusion patients with and without ICAS (12, 13). To date, no study has focused on prognostic factors related to rescue therapies, such as emergent angioplasty and stenting, in patients with acute posterior circulation LVO. Accordingly, we aimed to investigate clinical outcomes and predictors of favorable functional outcomes after EVT, including emergent angioplasty and stenting, in patients with acute ICAS-related occlusions in vertebrobasilar arteries.

## Materials and methods

### Patients

From February 2012 to December 2022, a total of 157 consecutive patients with acute LVO in the posterior circulation underwent EVT at a regional comprehensive stroke center. Of these, 46 patients with an occlusion in the basilar artery or an intracranial segment of the vertebral artery, due to underlying ICAS, underwent emergent angioplasty and/or stenting after a first-line mechanical thrombectomy. The study period was divided into two periods: earlier (from February 2012 to July 2017) and later (August 2017 to December 2022). We analyzed the clinical and radiologic data of these 46 patients, including demographic features, cerebrovascular risk factors, occlusion site, admission blood glucose level, admission score on the National Institutes of Health Stroke Scale (NIHSS), posterior circulation Alberta Stroke Program Early CT Score (pc-ASPECTS), use of intravenous thrombolytic agents, use of intra-arterial or intravenous antiplatelet drugs, time to procedure, reperfusion status, and post-procedural hemorrhagic complications, such as subarachnoid hemorrhage, parenchymal hemorrhage, or symptomatic hemorrhage. Patient data were prospectively collected in a stroke database, and retrieved for retrospective analysis. Hyperlipidemia was defined as hypercholesterolemia (total cholesterol ≥ 220 mg/dL, low-density lipoprotein ≥ 160 mg/dL, or triglyceride ≥ 150 mg/dL) or use of cholesterol-lowering medication. The blood lipid levels were determined on the day after admission in a fasting state (14). Admission hyperglycemia was defined as the first serum glucose on admission ≥ 140 mg/dL (15). The institutional ethics committee approved this retrospective analysis and waived the requirements for informed consent on the basis of the study design.

On admission, patients were neurologically evaluated by a stroke neurologist based on the NIHSS. All patients underwent non-enhanced computed tomography (CT) and/or magnetic resonance imaging (MRI) before the EVT. The inclusion criteria for the EVT were as follows: (1) presentation within 12 h of symptom onset or the last time the patient was observed in a well state; (2) baseline NIHSS score ≥ 4; (3) no intracranial hemorrhage detected on a cranial CT or MRI; (4) occlusion of the basilar artery or intracranial segment of the vertebral artery on catheter angiography; and (5) a pre-stroke modified Rankin Scale (mRS) score ≤ 3. The pc-ASPECTS was assessed, based on the pretreatment CT or diffusion-weighted MRI (16).

### Endovascular therapy

Before the EVT, written informed consent for an EVT was obtained from a family member for each patient. The EVT was performed under local anesthesia. In cases of agitation, an intravenous bolus of midazolam was given, and repeated, when necessary. The initial treatment was a mechanical thrombectomy, such as a stent-retriever thrombectomy, a contact aspiration thrombectomy, or a combination technique (e.g., simultaneous use of stent retriever and aspiration catheter). When severe (≥70%) underlying ICAS was detected on the follow-up angiography after the thrombectomy, a rescue therapy was performed, including intracranial angioplasty and/or stenting. The degree of arterial stenosis was determined according to Warfarin Aspirin Symptomatic Intracranial Disease criteria (17). Intracranial angioplasty was performed with a Gateway balloon catheter (Stryker Inc., Fremont, CA, USA). The diameter of the balloon was 80% of the normal diameter of the vessel just distal to the stenotic lesion. The balloon was inflated slowly, 1 or 2 times, with a screw-type pressure inflation device at 4 to 6 atm for 30–60 s. After intracranial angioplasty, at the operator’s discretion, intracranial stenting was performed with a Wingspan stent (Stryker Inc., Fremont, CA, USA) or Solitaire stent (Covidien, Irvine, CA, USA). Intra-arterial and/or intravenous glycoprotein IIb/IIIa inhibitor was administered after angioplasty/stenting, at the operator’s discretion. Patients received aspirin and clopidogrel orally after the procedure, when there was no intracranial hemorrhage on an immediate post-treatment CT scan. Dual antiplatelet therapy was then continued for the next 3 months.

### Outcome measures

Reperfusion status was assessed on a final angiogram according to the modified Treatment In Cerebral Ischemia grade, and successful reperfusion was defined as a grade of 2b or 3 (18). The arterial occlusion site was categorized as either a basilar or intracranial vertebral artery. The mRS was used to evaluate the functional outcome at 90 days after the EVT. An mRS of 0 to 3 was considered a favorable outcome. Post-treatment CT scans were evaluated to identify intracranial hemorrhages according to the Heidelberg bleeding classification (19). Accordingly, a symptomatic intracranial hemorrhage was defined as any hemorrhage associated with clinical evidence of neurological worsening, where the hemorrhage was judged to be the principal cause of neurologic decline. For patients in acceptable conditions, a follow-up brain CT angiography (CTA) was performed within 3–7 days after the EVT to evaluate the patency of the treated vessel. Early arterial reocclusion was defined as a discrete discontinuation in an arterial contrast column within the treated artery on a follow-up CTA (20). Two neuroradiologists that were blinded to the procedure assessed angiographic and CT images retrospectively, and conclusions were drawn by consensus.

### Statistical analysis

Continuous variables are expressed as the median and interquartile range (IQR). Categorical variables are expressed as the count (n) and percentage (%). First, the relationship between each clinical or procedural characteristic and the 90-day outcome was determined. Categorical and binary variables were compared with Pearson’s chi-square test or Fisher’s exact test. Continuous variables were compared with the Mann–Whitney U test. Second, multivariate binary logistic regression analyses were performed to identify independent predictors of favorable outcome (i.e., 90-day mRS = 0–3). Third, the association between early reocclusion detected on a CTA and the 90-day outcome was determined in a subgroup analysis that included only patients that underwent a follow-up CTA. The variables included in the logistic regression models were those with *p* < 0.05 in the univariate analysis. Results of logistic regression analyses are expressed as the odds ratio (OR) and 95% confidence interval (CI). All statistical analyses were performed with IBM SPSS Statistics for Windows, version 27 (IBM Corp., Armonk, NY, USA). *p*-values <0.05 were considered significant.

## Results

This study included 46 patients (56.5% male; median age 71; age range, 47–88 years) that underwent emergent angioplasty/stenting for ICAS-related occlusions in the vertebrobasilar arteries. The baseline and procedural characteristics of patients are presented in Table 1. The NIHSS scores ranged from 2 to 24 with a median of 11 (interquartile range [IQR], 7–20) at the time of admission. There were no missing data for patient characteristics and treatment outcomes in our study.

**Table 1:** Characteristics of patients with and without favorable outcomes.

Characteristic	All patients (*n* = 46)	Favorable outcome (mRS 0–3, *n* = 21)	Unfavorable outcome (mRS 4–6, *n* = 25)	*p* value
Age (years)	71 (61–78)	63 (55–74)	73 (67–78)	0.77
Sex (male)	26 (56.5)	13 (61.9)	13 (52)	0.5
The study period				0.264
Earlier	20 (43.5)	11 (52.4)	9 (36)	
Later	26 (56.5)	10 (47.6)	16 (64)	
Vascular risk factors				
Hypertension	40 (87)	19 (90.5)	21 (84)	0.516
Diabetes mellitus	19 (41.3)	6 (28.6)	13 (52)	0.108
Hyperlipidemia	14 (30.4)	11 (52.4)	3 (12)	0.004
Smoking	13 (28.3)	8 (38.1)	5 (20)	0.175
Atrial fibrillation	3 (6.5)	1 (4.8)	2 (8)	0.658
Coronary artery disease	4 (8.7)	0	4 (16)	0.055
Previous stroke or TIA	5 (10.9)	3 (14.3)	2 (8)	0.495
Occlusion site				0.023
Basilar artery	34 (73.9)	12 (57.1)	22 (88)	
Vertebral artery	12 (26.1)	9 (42.9)	3 (12)	
Stenting/Angioplasty				0.027
Stenting	32 (69.6)	11 (52.4)	21 (84)	
Angioplasty alone	14 (30.4)	10 (47.6)	4 (16)	
Baseline NIHSS	10.5 (7–20)	8 (5–15.5)	14 (9–21.5)	0.013
Admission hyperglycemia	29 (63)	10 (47.6)	19 (76)	0.047
pc-ASPECTS	7 (6–8)	8 (7–8)	7 (6–8)	0.2
Time to treatment (min)	327.5 (200–485)	240 (157.5–490)	340 (210–473)	0.408
Intravenous thrombolysis	12 (26.1)	7 (33.3)	5 (20)	0.305
Intra-arterial GPI	22 (47.8)	7 (33.3)	15 (60)	0.071
Intravenous tirofiban	20 (43.5)	8 (38.1)	12 (48)	0.5

Values are presented as n (%) or median (IQR), as indicated.

GPI, glycoprotein inhibitor; mRS, modified Rankin Scale; NIHSS, National Institute of Health Stroke Scale; pc-ASPECTS, posterior circulation Acute Stroke Prognosis Early Computed Tomography Score; TIA, transient ischemic attack.

**Figure 1:**
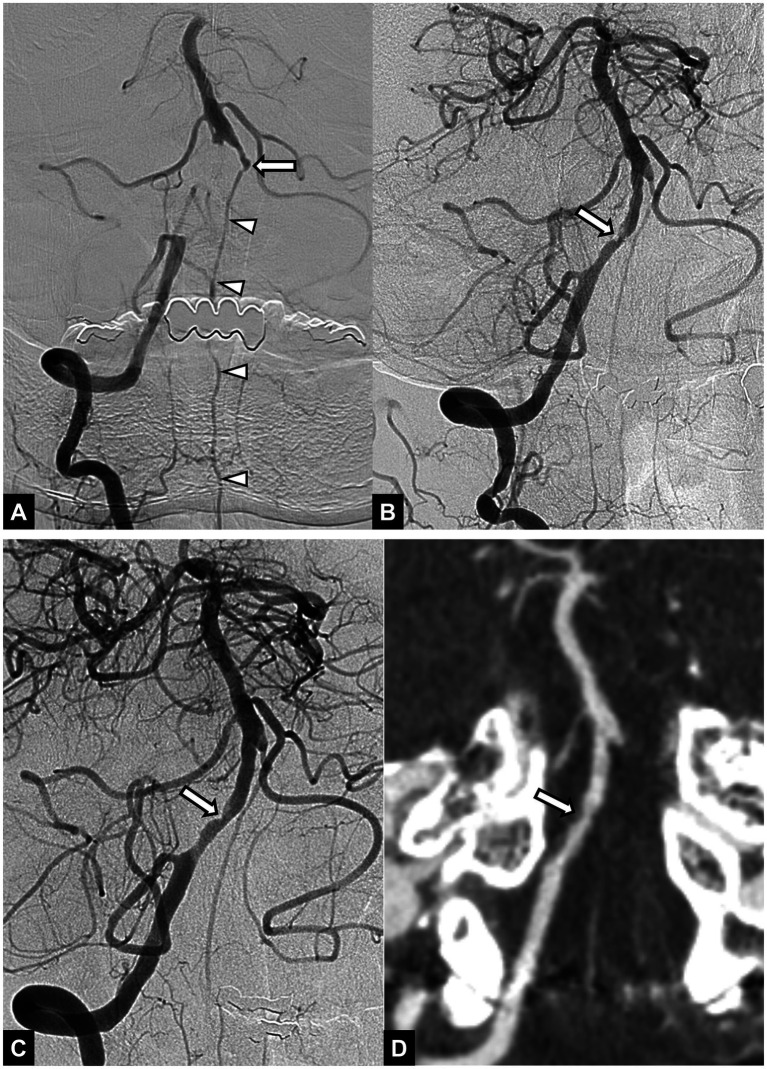
A 54-year-old male patient with acute stroke due to vertebral artery occlusion. **(A)** Initial angiogram shows an occlusion at the intracranial segment of the right vertebral artery. Basilar artery is opacified via collateral circulation from the anterior spinal artery (arrowheads). Note the chronic occlusion in the left vertebral artery (arrow). **(B)** Angiogram obtained after mechanical thrombectomy with a stent retriever shows successful recanalization with underlying focal irregular stenosis (arrow) in the right vertebral artery. **(C)** Final angiogram obtained after intracranial angioplasty shows successful reperfusion of the posterior circulation and remained stenosis (arrow) in the right vertebral artery. **(D)** Follow-up CT angiography obtained 48 h after endovascular treatment shows patent right vertebral artery with remained mild stenosis (arrow). The 3-month mRS score was 1.

**Figure 2:**
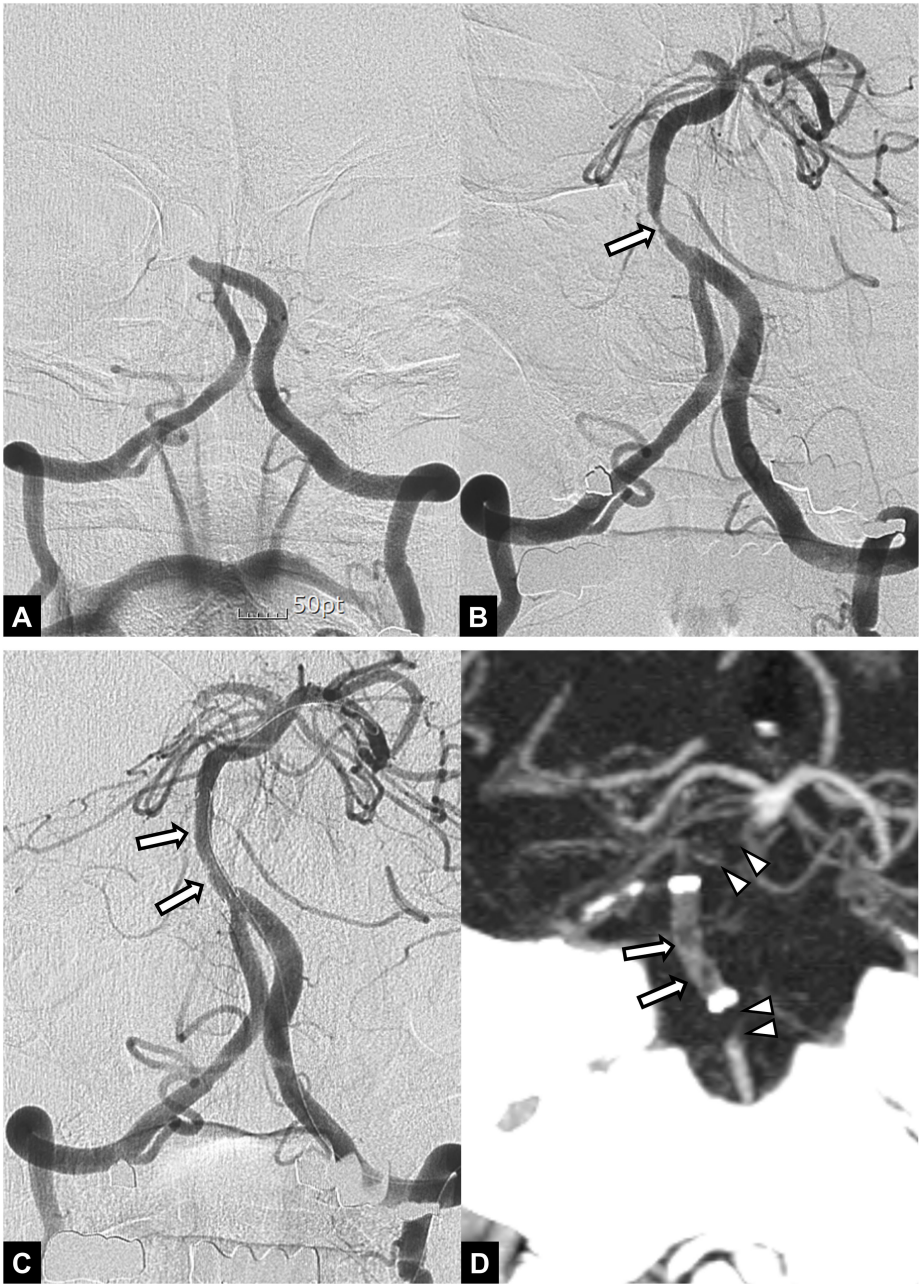
A 55-year-old male patient with acute stroke due to basilar artery occlusion. **(A)** Initial angiogram shows an occlusion at the proximal segment of the basilar artery. **(B)** Angiogram obtained after mechanical thrombectomy with a stent retriever shows recanalization of the basilar artery with underlying severe stenosis (arrow). Note accompanying occlusion in the right posterior cerebral artery. **(C)** Final angiogram obtained after angioplasty and stent implantation (arrows) to treat underlying stenosis shows successful recanalization of the basilar artery. **(D)** Follow-up CT angiography obtained 24 h after endovascular treatment shows occlusions in the implanted stent (arrows) and proximal and distal segment of the basilar artery (arrowheads). The patient died 2 days later.

For rescue therapy, 14 patients (30.4%) underwent intracranial angioplasty alone (Figure 1), 28 (60.9%) received intracranial stenting after angioplasty (Figure 2), and 4 (8.7%) received stenting alone. Stenting was performed with a Wingspan stent in 23 patients and Solitaire stent in 9 patients. Twenty-two patients (47.8%) received an intra-arterial infusion of glycoprotein IIb/IIIa inhibitor (Abxicimab, Reopro; Eli Lilly and Co., IN, USA; or tirofiban, Aggrastat; Merck, Whitehouse Station, NJ, USA) during the procedure, and 20 patients received an intravenous infusion of tirofiban after the procedure.

Overall, 95.7% (44/46) of patients achieved successful reperfusion after the EVT. In 2 patients (4.3%), a subarachnoid hemorrhage was detected on post-procedure CT scans. A parenchymal hemorrhage occurred in 6 patients (13%) and a symptomatic hemorrhage occurred in 1 patient (2.2%). At the time of discharge, the NIHSS scores ranged from 0 to 39 with a median of 7. The 90-day mortality rate was 23.9% (11/46). In-hospital mortality occurred in 4 of 11 patients. A favorable outcome (90-day mRS score 0 to 3) was achieved in 45.7% of patients (21/46). The rate of favorable outcomes was significantly higher among patients with hyperlipidemia than among those without hyperlipidemia (78.6% [11/14] vs. 31.3% [10/32], *p* = 0.004). Patients with admission hyperglycemia had significantly fewer favorable outcomes than those without admission hyperglycemia (34.5% [10/29] vs. 64.7% [11/17], *p* = 0.047). In patients with admission hyperglycemia, the level of blood glucose ranged from 142 to 265 mg/dL with a median of 167 mg/dL (IQR, 158–203 mg/dL). Patients with a favorable outcome had a significantly lower admission NIHSS compared to those with an unfavorable outcome (8 vs. 14, *p* = 0.013). Patients with basilar artery occlusions had favorable outcomes significantly less frequently than those with occlusions in the intracranial vertebral artery (35.3% [12/34] vs. 75% [9/12], *p* = 0.023). Patients treated with angioplasty alone had favorable outcomes significantly more frequently than those treated with stenting (71.4% [10/14] vs. 34.4% [11/32], *p* = 0.027). Patients with favorable outcomes and those with unfavorable outcomes were not significantly different, in terms of other baseline characteristics, intravenous thrombolysis, intra-arterial or intravenous infusion of glycoprotein inhibitor, or the rates of successful reperfusion and hemorrhagic complications. In 32 patients who received intracranial stenting, the type of stent (Wingspan versus Solitaire) was not associated with functional outcomes (*p* = 0.445).

A multivariate binary logistic regression analysis that included the study period (earlier vs. later), hyperlipidemia, admission hyperglycemia, admission NIHSS, occlusion site (basilar artery vs. vertebral artery), and angioplasty alone treatment (vs. intracranial stenting) revealed following independent predictors of a 90-day favorable outcome: hyperlipidemia (OR = 7.866, 95% CI: 1.093–56.590, *p* = 0.040), admission hyperglycemia (OR = 0.144, 95% CI: 0.023–0.914, *p* = 0.040), basilar artery occlusion (OR = 0.086, 95% CI: 0.008–0.907, *p* = 0.041), and treatment with angioplasty alone (OR = 9.779, 95% CI: 1.022–93.525, *p* = 0.048) (Table 2).

**Table 2:** Univariate and multivariate logistic regression analyses for predictors of a 90-day favorable outcome.

Predictors of favorable outcome	Unadjusted OR	95% CI	*p* value	Adjusted OR	95% CI	*p* value
Hyperlipidemia	8.067	1.837–35.413	0.006	7.866	1.093–56.590	0.040
Admission hyperglycemia	0.287	0.082–1.007	0.051	0.144	0.023–0.914	0.040
Baseline NIHSS score, per 1-point increase	0.891	0.807–0.984	0.023	–	–	–
Occlusion site (Basilar vs. vertebral)	0.182	0.041–0.802	0.024	0.086	0.008–0.907	0.041
Angioplasty alone treatment	4.773	1.213–18.781	0.025	9.779	1.022–93.525	0.048
Study period (Earlier vs. later)	0.511	0.157–1.670	0.267			

CI, confidence interval; NIHSS, National Institute of Health Stroke Scale; OR, odds ratio.

Of the 46 patients, 42 underwent follow-up CTA. Four patients could not undergo follow-up CTA due to poor medical condition. Early reocclusion of the treated artery occurred in 8 patients (19%) (Figure 2). Among these 8 patients, 6 were treated with angioplasty, followed by stenting, and 2 were treated with angioplasty alone; these proportions were not significantly different (21.4% [6/28] vs. 14.3% [2/14], *p* = 0.697). In a subgroup analysis that included only patients with follow-up CTA (*n* = 42), patients with early reocclusions had unfavorable outcomes (mRS score of 4–6) significantly more frequently than those without reocclusions (87.5% [7/8] vs. 41.2% [14/34]; OR = 10.0, 95% CI: 1.104–90.593) (Table 3). In addition, mortality at 90 days occurred more frequently among patients with early reocclusion than among those without reocclusions (50% [4/8] vs. 8.8% [3/34]; OR = 10.333, 95% CI: 1.668–63.999). However, patients with and without early reocclusions were not significantly different, in terms of whether they received an intra-arterial administration or intravenous infusion of glycoprotein inhibitors.

**Table 3:** Comparison of outcomes between patients with and without early reocclusion on CTA in 42 patients.

	All patients (*n* = 42)	Patients with early reocclusion (*n* = 8)	Patients without early reocclusion (*n* = 34)	*p* value
Admission hyperglycemia	28 (66.7)	8 (100)	20 (58.8)	0.026
Use of IA GPI	21 (50)	6 (75)	15 (44.1)	0.116
Use of IV GPI	19 (45.2)	4 (50)	15 (44.1)	0.764
Angioplasty alone treatment	14 (33.3)	2 (25)	12 (35.3)	0.578
Favorable outcome	21 (50)	1 (12.5)	20 (58.8)	0.018
Symptomatic hemorrhage	1 (2.4)	0	1 (2.9)	0.623
Mortality	7 (16.7)	4 (50)	3 (8.8)	0.005

GPI, glycoprotein inhibitor; IA, intraarterial; IV, intravenous; mRS, modified Rankin Scale.

Patients treated with angioplasty alone had fewer basilar artery occlusions (57.1% [8/14] vs. 81.3% [26/32], *p* = 0.087) and a lower rate of early reocclusion (14.3% [2/14] vs. 21.4% [6/28], *p* = 0.697), compared to those treated with stenting. However, these differences were not statistically significant.

## Discussion

Our results suggested that angioplasty and stenting could achieve high rates of successful reperfusion and favorable outcome in patients with acute vertebrobasilar artery occlusions due to an underlying ICAS. However, the optimal treatment strategy for these patients has yet to be determined. In our study, all patients were treated with angioplasty or stenting as a rescue therapy after an initial mechanical thrombectomy. Successful reperfusion was achieved in 96%, and favorable functional outcomes (90-day mRS = 0–3) were achieved in 46% of patients. The rate of successful reperfusion in our study seems higher than those reported in recent randomized trials which ranged from 71 to 88% (21–23). However, direct comparison is inappropriate because patient inclusion and exclusion criteria are different from each other. The rate of 90-day mRS = 0 to 3 (46%) in our study was higher than the 24–38% reported in the medical therapy only group and comparable to the 42–46% reported in the thrombectomy group in recent randomized trials that investigated EVT for posterior circulation LVO (15–18). Of note, 40 and 55% of patients in the thrombectomy groups of both the ATTENTION (Endovascular Treatment for Acute Basilar-Artery Occlusion) trial and the BAOCHE (Basilar Artery Occlusion Chinese Endovascular) trial received intracranial angioplasty or stenting after a failed thrombectomy (21, 24). That was not surprising, considering that ICAS is highly prevalent among Chinese patients with posterior circulation LVOs. The results of our study, taken together with recent successful randomized trials, suggested that angioplasty and stenting comprise an effective rescue therapy for an underlying ICAS in patients with acute vertebrobasilar artery occlusion. In addition, the mortality rate (24%) in our study was lower than the 31–38% reported in the thrombectomy group and the 38–55% reported in the control group of recent randomized trials (21–24). This was likely related to the fact that the patients who had extensive brain stem infarction were excluded in our study. Thus, the median admission NIHSS score in our study (10.5) was lower than those (20–24) reported in recent trials. A recent systematic review and meta-analysis conducted by Almallouhi et al. also confirmed the efficacy of rescue therapy including angioplasty and stenting, in patients with ICAS-related LVO (25). In their analyses, patients with ICAS-related LVO that received rescue therapy had an increased probability of a favorable outcome (OR = 3.19) and a reduced probability of mortality (OR = 0.35), compared to patients that did not receive rescue therapy.

To date, data are scarce regarding the predictors of functional outcomes after an EVT in patients with ICAS-related LVOs. In a recent retrospective study by Baek et al. on patients with ICAS-related LVOs in the anterior circulation, a poor outcome (90-day mRS = 3–6) could be independently predicted by a history of previous stroke/transient ischemic attack, a high admission NIHSS, and an early reocclusion of the treated artery (26). To our knowledge, the present study was the first to identify factors associated with functional outcomes after an emergent angioplasty and/or stenting in patients with ICAS-related LVOs in vertebrobasilar arteries. Our study demonstrated that hyperlipidemia, admission hyperglycemia, occlusion site, and angioplasty alone treatment could be associated with a 90-day favorable outcome in these patients.

Hyperlipidemia is a well-known risk factor for atherosclerosis and ischemic stroke. Interestingly, several studies have found that hyperlipidemia was paradoxically associated with better functional outcomes and lower risk of hemorrhagic transformation after an EVT, in patients with acute LVO (15, 27). A recent meta-analysis also showed that hyperlipidemia was correlated with good outcomes after an EVT in posterior circulation LVO (28). Added to these previous findings, our results suggested that hyperlipidemia also had paradoxical protective effect on outcomes after EVT in patients with ICAS-related LVOs. Although the underlying mechanism remains unclear, several mechanisms might explain this observation. It has been suggested that systemic circulatory cholesterol may penetrate the damaged blood brain barrier in ischemic conditions and enhance the repair and remyelination of ischemic penumbral tissues (15). Cholesterol also may limit an infarct extension by serving as a buffer for free radicals released during an ischemic injury (29). Other plausible mechanisms include mitochondrial adaptations to chronic hyperlipidemia, the anti-inflammatory activity of cholesterol, and the neuroprotective effects of statins, given as treatment for hyperlipidemia (29, 30).

Our study showed that admission hyperglycemia was independently associated with lower odds (adjusted OR = 0.145) of a favorable outcome in patients with ICAS-related LVO treated with angioplasty and stenting. This observation was consistent with previous reports that demonstrated that admission hyperglycemia was associated with worse functional outcomes and increased risk of symptomatic hemorrhage and mortality in patients with LVOs treated with a thrombectomy (16, 31–33). Data from the MR CLEAN registry also showed that increased admission glucose was associated with a poor functional outcome and elevated risk of symptomatic hemorrhage after a thrombectomy (16). Our study differed from previous studies, because large artery atherosclerosis was the major stroke etiology (100%), and all patients underwent rescue angioplasty and/or stenting in addition to a thrombectomy. A previous Chinese multicenter registry study by Gu et al. showed that admission hyperglycemia was correlated with increased odds of symptomatic intracranial hemorrhage (OR = 3.24) and a poor 90-day outcome (OR = 1.91) among patients with vertebrobasilar artery occlusion treated with EVT (32). In their study, 73% of patients with admission hyperglycemia had large artery atherosclerosis as the stroke etiology. Taken together, our study results and those of Gu’s study suggested that hyperglycemia had a detrimental effect on EVT outcomes, regardless of the stroke etiology and the EVT method.

The negative effect of hyperglycemia on outcomes in patients with acute ischemic stroke may be attributed to several mechanisms. Hyperglycemia worsens cortical intracellular brain acidosis and mitochondrial function in the ischemic penumbra; these conditions promote the progression of ischemic penumbra to infarction (34). In addition, hyperglycemia may aggravate a reperfusion injury by increasing oxidative stress and blood–brain-barrier permeability. These conditions increase the risk of brain edema, hemorrhagic transformation, and infarct extension (35). Although previous studies showed an association of admission hyperglycemia with poor outcomes in acute ischemic stroke, supporting evidence on the use of glucose-lowering drug in acute stroke patients with hyperglycemia is still lacking (36–39). The GIST-UK and SELESTIAL trials found that intensive glycemic control did not significantly improve neurological prognosis or affect the growth of cerebral infarction in acute stroke patients with hyperglycemia (36, 37). Instead, these studies reported delays in therapy initiation and a higher incidence of hypoglycemia in such patients (36–39).

Our findings suggested that angioplasty alone might be a good option for rescue therapy in ICAS-related vertebrobasilar artery occlusions. In the current study, a favorable outcome occurred more frequently among patients that received angioplasty alone than those treated with stenting (71.4% vs. 34.4%, *p* = 0.027). This finding may partly be attributed to the lower rates of both basilar artery occlusion (57.1% vs. 81.3%, *p* = 0.087) and early reocclusion (14.3% vs. 21.4%, *p* = 0.697) in the angioplasty alone group compared to the stenting group. Our multivariate analysis showed that favorable outcome occurred significantly less frequently among patients with basilar artery occlusion than among those with intracranial vertebral artery occlusions. This difference may be due to the fact that brain stem infarctions occur more commonly in basilar artery occlusions than in vertebral artery occlusions. Moreover, previous studies have suggested that early reocclusion after an EVT may be associated with early neurological deterioration, poor functional outcome, and increased mortality (20, 26). In the present study, we found that, after adjusting for the occlusion site and the admission NIHSS score, the use of angioplasty alone was independently associated with a favorable outcome (OR = 7.745). This observation was consistent with a recent retrospective study by Luo et al., which showed that favorable outcome rates (mRS = 0–3 at 90 days) were comparable among patients treated with angioplasty alone, balloon mounted stents, and self-expanding stents (32.2% vs. 29.4% vs. 35.3%) (40). In that study, all patients had basilar artery occlusions, and the proportion of patients that had ICAS was not reported. Taken together, our results and Luo’s results suggested that angioplasty alone may be at least equivalent to stenting for treating ICAS-related occlusions in vertebrobasilar arteries. Moreover, angioplasty alone has several theoretical advantages over stenting. Compared to intracranial stenting, angioplasty alone may lower the risk of both perforator artery occlusions and acute thrombosis in the treated arteries (3). In addition, angioplasty alone could be a better option than stenting when adequate antiplatelet therapy is not possible, due to concerns about intracranial hemorrhage (3, 40). Based on the results of our study and the above considerations, it would be reasonable to reserve emergent stenting for circumstances where arterial dissection or progressive reocclusion occurs after angioplasty. Stenting may be omitted if the patient had long segmental stenosis or tortuous proximal vessel or hypoplastic parent vessel.

The major concern when performing angioplasty and stenting in patients with ICAS-related LVO is early reocclusion of treated arteries, which is likely induced by platelet aggregation on the injured endothelium. Thus, adequate antiplatelet therapy may be essential in patients undergoing emergent angioplasty/stenting for ICAS-related LVO. Recently, Baek et al. showed that the use of intravenous continuous infusion of glycoprotein IIb/IIIa receptor inhibitor is promising to prevent early reocclusion of treated arteries, with no increased risk of hemorrhage after emergent angioplasty/stenting in such patients (26). Evidence on this topic is scarce, and further randomized controlled trials are needed in the future.

This study had some limitations. The main limitations were the small sample size and the retrospective, non-randomized study design. Additionally, early reocclusion was not included in the binary logistic regression analysis, because early reocclusion could not be evaluated in 4 patients that lacked a follow-up CTA. Finally, the administration of glycoprotein inhibitor via an intra-arterial or intravenous route was inconsistent in our study; this inconsistency might have affected the occurrence of early reocclusion. However, the use of glycoprotein inhibitors was not associated with the occurrence of early reocclusion in our study.

In conclusion, our results indicated that emergent angioplasty and/or stenting could yield high rates of successful reperfusion and favorable functional outcomes in patients with ICAS-related occlusions in vertebrobasilar arteries. Our study also demonstrated that hyperlipidemia and treatment with angioplasty alone were independently associated with favorable outcomes, in contrast, admission hyperglycemia and basilar artery occlusion with unfavorable outcomes in these patients.

## Data Availability

The original contributions presented in the study are included in the article/supplementary material, further inquiries can be directed to the corresponding author.
